# Regulation of *TTN* as a mechanism of and treatment for heart failure

**DOI:** 10.1172/JCI189335

**Published:** 2025-02-17

**Authors:** Dominic E. Fullenkamp

**Affiliations:** Center for Genetic Medicine, Bluhm Cardiovascular Institute, Northwestern University Feinberg School of Medicine, Chicago, Illinois, USA.

## Abstract

Truncation variants in the gene *TTN* encoding titin are the most common cause of familial dilated cardiomyopathy (DCM), with both haploinsufficiency and “poison peptide” implicated as contributory mechanisms of disease. In this issue of the *JCI*, Kim et al. identify a highly conserved enhancer element approximately 500 bp downstream of the transcriptional start site of *TTN* in intron 1, which they demonstrated to be critical in regulating *TTN* expression. This work helps to further clarify the relative role of haploinsufficiency in *TTN*-related DCM and provides a potential target for therapies aimed at treating *TTN*-related DCM.

## *TTN* truncations increase the risk of DCM

**Figure 1:**
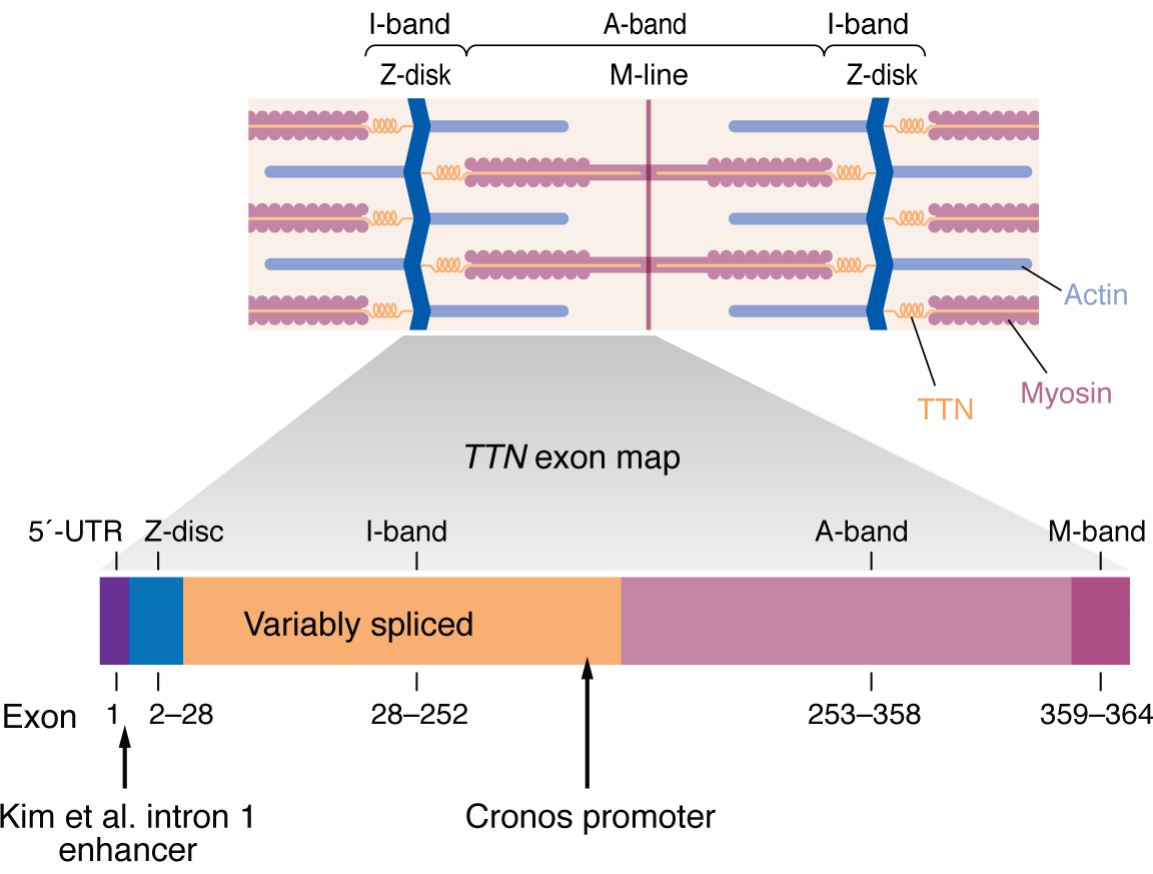
*TTN* encodes the giant myofilament protein TTN that spans the full half-sarcomere. TTN spans the Z-disc to the M-line in the half-sarcomere and has a structural role. The intron 1 enhancer identified by Kim et al. is approximately 500 bp downstream of the *TTN* transcriptional start site (18), with the translational start site found in exon 2. The I-band is variably spliced to give the dominant cardiac isoforms N2B and N2BA. An alternative promoter near the A/I junction gives rise to the Cronos isoform (3).

Dilated cardiomyopathy (DCM) is a common clinical disease with a prevalence that has been estimated to be 1:250, with familial causes estimated to account for 23% of DCM cases (1). Heterozygous truncation variants in the gene *TTN* encoding titin are the leading cause of genetically mediated cardiomyopathy, accounting for up to 25% of familial DCM cases and 18% of sporadic cases (2). TTN spans the Z-disc (N-terminal) to the M-band (C-terminal) and contains an A-band domain and a spring-like I-band domain connecting the full half sarcomere. *TTN* is composed of 364 exons and codes for the largest known protein TTN with the dominant human heart isoforms N2BA (approximately 3.3–3.5 MDa) and N2B (approximately 3.0 MDa), and the shorter isoform Cronos (approximately 2.2 MDa), which arises from an alternative promoter at around the A/I junction (3, 4) (Figure 1). TTN plays critical roles in sarcomere development and patterning, as well mechanical and signaling roles (5).

*TTNtvs* that are found in the A-band or other highly expressed exons in the heart are most associated with DCM and are generally dominantly inherited (3). Like many other forms of genetic heart disease, there is incomplete age-dependent penetrance with variable expressivity in patients with *TTNtvs* (1). Beyond this, studies of patients with peripartum cardiomyopathy (6), chemotherapy-related cardiomyopathy (7), alcohol-related cardiomyopathy (8), and early-onset atrial fibrillation (9) have all been shown to be enriched in patients harboring *TTNtvs*. While some *TTNtvs* are highly penetrant in certain families, a two- or multi-hit model in which other risk factors are additive, in addition to nonmonogenic genetic background effects, likely account for variable penetrance and expressivity of the disease. In the context of atrial fibrillation, the presence of a *TTNtv* has been shown to be additive to polygenic risk, with the highest penetrance of atrial fibrillation in those with *TTNtv* and high polygenic risk (10).

## Pathogenesis of *TTNtv-*mediated DCM

Two nonmutually exclusive hypotheses have been developed to explain DCM pathogenesis for *TTNtvs*, namely, haploinsufficiency and “poison peptide.” Full-length TTN is decreased in *TTNtv* DCM hearts compared with non-*TTNtv* DCM hearts, and truncated TTN proteins have been shown to incorporate into sarcomeres in patients with DCM (11–14). Using explanted DCM hearts, Formin et al. demonstrated stable expression of truncated TTN proteins that localized to intracellular aggregates (12). They showed reduced content of full-length TTN and were able to functionally rescue a *TTNtv* human induced pluripotent stem cell (hiPSC) engineered heart muscle model using proteosome inhibition, which correlated with increased full-length TTN content (12). Romano et al. developed an hiPSC-cardiomyocyte (hiPSC-CM) microtissue model that showed deficits in contractility in *TTN* A-band truncation tissue along with the presence of TTN truncation proteins (15). They also noted partial recovery of contractile loss when an I-band truncation was edited in *cis*, concluding that there were contributions from poison peptides and haploinsufficiency (15). In a mouse model of an A-band *Ttntv*, homozygous *Ttntv* mice demonstrated embryonic lethality, while heterozygous *Ttntv* mice showed normal cardiac function and expression of Ttn truncated proteins (16). The mice only developed left ventricular dysfunction compared with wild-type mice after treatment with angiotensin II. An excellent and detailed Commentary in the *JCI* by Hinson and Campbell describes the current literature in this area (17).

## Is *TTN* haploinsufficiency sufficient to cause disease?

In the vast majority of clinically identified patients with pathogenic *TTN* variants, there is the possibility of producing truncated TTN protein. In this issue of the *JCI*, Kim et al. identified an enhancer in intron 1 of *TTN* found approximately 500 bp downstream of the transcription start site (Figure 1) that is highly conserved with NKX2-5 and MEF2 transcription factor binding motifs (18). Using the assay for transposase-accessible chromatin sequencing (ATAC-Seq), Kim and co-authors demonstrated increased chromatin accessibility during CM differentiation in hiPSC-CMs. They deleted this region in mice and showed embryonic lethality for homozygous mice. The heterozygous mice, while phenotypically normal by echocardiography, demonstrated decreased *Ttn* expression and that regulation was in *cis*. The authors further demonstrated that compound heterozygous mice with deletion of the enhancer in *trans* with a *Ttntv* resulted in embryonic lethality, further confirming the importance of the enhancer (18).

Kim and colleagues identified a smaller region (E1) of this enhancer with predicted NKX2-5 and MEF2 binding sites and, using hiPSC-CMs, showed that this region stimulated GFP expression through a GFP reporter assay. Deletion of E1 in heterozygous hiPSC-CMs decreased *TTN* expression, with a substantial reduction in homozygous hiPSC-CMs. Sarcomere density and structure were impaired in the heterozygous E1 deletion, with more marked alterations in the homozygous deletion. Interestingly, the microtissues generated from the E1 deletion did not show a reduction in active force production, while the homozygous deletion of E1 had roughly a 50% reduction in force (18). This finding contrasts with prior work using hiPSC-CM microtissue models of *TTNtvs*, which have consistently shown decreased force production in the heterozygous state (5, 15). The preservation of force may be related to the absence of a truncated TTN product, as this deletion specifically targets a regulatory region of the gene. Kim and colleagues went on to identify a rare variant in E1 in the predicted myocyte enhancer factor 2 (MEF2) binding site (chr2:178,806,843T>C) from a patient with unexplained DCM (18). Engineering this variant into hiPSC-CMs in biallelic fashion resulted in reduced *TTN* expression and reduced TTN protein. While reduced force was not seen in the heterozygous E1 deletion hiPSC-CM microtissues, Kim and co-authors did see mild but significantly reduced force production in the homozygous chr2:178,806,843T>C hiPSC-CM microtissues (18).

## Future directions and clinical implications

Heterozygous *Ttntv* mice have a minimal phenotype at baseline (16), which contrasts with most *TTNtv* hiPSC-CM models that show baseline functional deficits (5). Prior studies indicate that *Ttntv* mice have normal TTN levels at baseline (16), whereas DCM *TTNtv* hearts have deficiencies in TTN protein (11, 12). These observations were made using materials from end-stage explanted hearts, and the clinical rationale and equipoise are insufficient to subject patients with *TTNtvs* to endomyocardial biopsies earlier in the disease. Although useful, it is unclear what stage of disease hiPSC-CM models represent. Furthermore, heterozygous *TTNtv* carriers often present with heart failure in the setting of other conditions like pregnancy, chemotherapy, or even extensive alcohol use. Whether TTN protein content shifts during these physiological stressors is unknown. It is known that *TTN* splicing shifts during heart failure. It is also clear that TTN truncated proteins are integrated into the myofibril, altering sarcomere function. However, how these changes influence disease pathogenesis remains an open question that hiPSC-CM–derived cardiac models are likely to play an important role in further elucidating.

The work of Kim et al. (18) suggests a pathogenic contribution of the intron 1 *TTN* rare variant and highlights a major limitation of current clinical testing, namely that this variant would have never been found with current clinical panel testing. It also suggests the lines between monogenic and polygenic disease will become furthered blurred as these sorts of regulatory variants are increasingly identified. Outside of engineering hiPSC lines, there are limited tools to use in adjudicating the pathogenicity of these variants. Rare noncoding variants are not assessed by gene panel sequencing, nor are they included in arrays evaluating polygenic risks. It would be interesting to know whether the mouse model in Kim et al. (18) would develop cardiomyopathic features using a stressor like angiotensin II, especially if, that were compared directly with a *Ttntv* mouse on the same genetic background. This sort of model would help to better adjudicate the relative contributions of haploinsufficiency and the “poison peptide” mechanisms of disease.

*TTN* upregulation through a CRISPR activation strategy has been put forward as a potentially viable approach for virtually all patients with *TTNtvs,* as engineered tissue models showed restoration of wild-type TTN protein content and improvement in force (19). However, in addition to full-length TTN protein, TTN truncated proteins were also upregulated, and it is unclear whether there might be potential detrimental long-term consequences of upregulation of these truncated proteins. The work by Kim et al. identifies an additional molecular target region that might be used in a CRISPR activation strategy for *TTN* upregulation (18). Given that “poison peptides” likely play some role in the pathogenicity of *TTNtv* DCM, whether through direct effects on the myofilament and/or through accumulation of cellular aggregates, there is also a potential downside to this strategy. The question of whether such strategies in *TTNtv* DCM are helpful or potentially harmful awaits further investigation. While gene replacement seems unlikely to become a viable strategy for *TTN*-related disease, given the tremendous size of *TTN*, gene editing and exon skipping are also potentially viable strategies (15, 20). The regulatory landscape of the FDA Modernization Act 2.0 and hiPSC-CM models may begin to give us a framework of how to evaluate these therapeutics for patients with *TTNtv*, most of whom have rare and even unique mutations that would be difficult to assess in a conventional clinical trial. Given the variable penetrance and expressivity of the *TTN* gene in cardiomyopathy, which is often responsive to guideline-directed medical therapy for heart failure, identification of the point during the disease course when there is clinical equipoise for trials related to gene editing and regulation merits careful scrutiny.

## Version 1 02/17/2025

Electronic publication
